# Predictive value of the inconsistency between the residual and post-PCI QFR for prognosis in PCI patients

**DOI:** 10.3389/fcvm.2024.1297218

**Published:** 2024-04-17

**Authors:** Lihua Chen, Jiaxin Zhong, Ruijin Hong, Yuxiang Chen, Beilei Li, Laicheng Wang, Yuanming Yan, Lianglong Chen, Qin Chen, Yukun Luo

**Affiliations:** ^1^ Department of Cardiology, Fujian Medical University Union Hospital, Fuzhou, Fujian, China; ^2^ Fujian Institute of Coronary Heart Disease, Fujian Medical University Union Hospital, Fuzhou, Fujian, China; ^3^ Fujian Heart Medical Center, Fujian Medical University Union Hospital, Fuzhou, Fujian, China; ^4^ Changle District People's Hospital Cardiovascular Department, Fuzhou, Fujian, China

**Keywords:** quantitative flow ratio, percutaneous coronary intervention, coronary artery disease, target vessel failure, prognostic value

## Abstract

**Introduction:**

To investigate the prognostic value of the consistency between the residual quantitative flow ratio (QFR) and postpercutaneous coronary intervention (PCI) QFR in patients undergoing revascularization.

**Methods:**

This was a single-center, retrospective, observational study. All enrolled patients were divided into five groups according to the ΔQFR (defined as the value of the post-PCI QFR minus the residual QFR): (1) Overanticipated group; (2) Slightly overanticipated group; (3) Consistent group; (4) Slightly underanticipated group; and (5) Underanticipated group. The primary outcome was the 5-year target vessel failure (TVF).

**Results:**

A total of 1373 patients were included in the final analysis. The pre-PCI QFR and post-PCI QFR were significantly different among the five groups. TVF within 5 years occurred in 189 patients in all the groups. The incidence of TVF was significantly greater in the underanticipated group than in the consistent group (*P* = 0.008), whereas no significant differences were found when comparing the underanticipated group with the other three groups. Restricted cubic spline regression analysis showed that the risk of TVF was nonlinearly related to the ΔQFR. A multivariate Cox regression model revealed that a ΔQFR≤ −0.1 was an independent risk factor for TVF.

**Conclusions:**

The consistency between the residual QFR and post-PCI QFR may be associated with the long-term prognosis of patients. Patients whose post-PCI QFR is significantly lower than the residual QFR may be at greater risk of TVF. An aggressive PCI strategy for lesions is anticipated to have less functional benefit and may not result in a better clinical outcome.

## Introduction

Percutaneous coronary intervention (PCI) is widely recognized in clinical practice to improve the symptoms and outcomes of patients with coronary artery disease (CAD) (1, 2). Despite the established benefits of PCI, certain patients who undergo successful PCI still experience adverse cardiovascular events (3). Conventional coronary angiography can provide information only on the contour of the culprit vessel and may not account for physiological dysfunction, which explains the unanticipated adverse events in patients undergoing successful revascularization (4).

Physiological assessments are of increasing importance because of their ability to provide functional information about target vessels and to optimize treatment strategies (5, 6). Fractional flow reserve (FFR) is a widely accepted physiological assessment technique and is considered the gold standard in revascularization procedures (7, 8). Although the FFR provides significant functional information, it is still underutilized in clinical practice due to the prolonged procedure time and invasive use of guidewires (9, 10). The quantitative flow ratio (QFR) has emerged as an alternative approach for deriving physiological parameters, with the advantages of having equivalent diagnostic value and being faster and more convenient than the FFR (11, 12).

The residual QFR is an essential indicator derived from the QFR computation procedure and can simulate the anticipated QFR value after successful revascularization based on pre-PCI angiographic images (13). Previous studies have reported that the residual QFR significantly correlates with the post-PCI FFR, especially in patients with suboptimal PCI results (14). Based on the residual QFR, a cardiologist can identify the major lesion from a functional perspective, further optimizing the PCI strategy. The residual QFR-guided PCI strategy was superior to angiographic guidance in reducing the 2-year incidence of target vessel failure (TVF) (15, 16). However, research into the predictive value of the residual QFR for adverse events is limited, particularly in patients whose residual QFR does not match their post-PCI QFR. Therefore, the present study aimed to further investigate the prognostic value of the residual QFR by exploring the correlation between the ΔQFR (defined as the value of the post-PCI QFR minus the residual QFR) and clinical outcomes.

## Materials and methods

### Study design

The present research was a single-center, retrospective, observational study. Consecutive patients who underwent PCI were recruited from January 1, 2016, to December 31, 2017, at Fujian Medical University Union Hospital. The QFR of the enrolled patients were retrospectively assessed at the different time points of the PCI procedure, and the patients were further divided into five groups according to the ΔQFR: (1) Overanticipated group: ΔQFR ≥ 0.1; (2) Slightly overanticipated group: 0 < ΔQFR < 0.1; (3) Consistent group: ΔQFR = 0; (4) Slightly underanticipated group: −0.1 < ΔQFR < 0; and (5) Underanticipated group: ΔQFR ≤ −0.1. The primary purpose of this study was to test the prognostic value of the consistency between the residual QFR and post-PCI for cardiovascular adverse events. This study was approved by the Ethics Committee of Fujian Medical University Union Hospital (No. 2020KY098).

The study population consisted of adult patients who underwent successful PCI, including patients with stable or unstable angina pectoris, non-ST elevation myocardial infarction (NSTEMI), or ST elevation myocardial infarction (STEMI), over 7 days. All enrolled patients met the requirements for QFR computation, which suggests that in all patients, at least one lesion with a percent diameter stenosis (DS%) between 50% and 90% is present in a coronary artery with a reference vessel diameter of ≥2.5 mm according to visual assessment. Patients were excluded if they had any of the following criteria: (1) acute myocardial infarction (AMI) within 7 days, (2) lack of follow-up data, or (3) inability to perform QFR computation, including patients who only had one coronary artery lesion with >90% stenosis and a TIMI grade <3; the interrogated lesion involving the myocardial bridge; severe overlap in the stenosed segment or severe tortuosity of any interrogated vessel; or poor angiographic image quality.

### PCI procedure and QFR computation

PCI was performed, and the stenting strategy was determined by an experienced cardiologist according to the ESC/EACTS guidelines at the time of enrollment (17). All patients received standard dual antiplatelet therapy for at least 12 months after successful revascularization. Rational medication was prescribed according to the clinical situation.

The QFR was computed using the AngioPlus system (Pulse Medical Imaging Technology Shanghai, China) according to standard operating procedures, which were performed by two independent investigators blinded to the clinical data. All coronary angiography images were transferred locally to the AngioPlus system. Angiographic images were recorded with an AngioPlus system at a rate of 15 frames/second. Two angiographic image runs, acquired at angles greater than or equal to 25 degrees, were transferred to the QFR system via the local network. Based on the reconstruction of the contoured vessels, the QFR value was computed using a contrast flow velocity model. The QCA information derived from the QFR analysis of the interrogated vessels consisted of the minimum lumen diameter (MLD), diameter stenosis percentage (DS%) and area stenosis percentage (AS%).

### Data collection and clinical outcomes

The biochemical indices and examination results, including low-density lipoprotein cholesterol (LDL-C), fasting blood glucose, creatinine, N-terminal pro brain natriuretic peptide (NT-proBNP), troponin I, left ventricular ejection fraction (LVEF), and E/E′, were recorded. E/E′ is the ratio of the peak mitral early filling velocity (E) to the early diastolic mitral annular velocity (E′), as an indicator of diastolic cardiac function.

Target vessel failure (TVF) was defined as a combination of cardiogenic death, target vessel-related myocardial infarction and target vessel revascularization (TVR) (18). TVR was defined as a repeat PCI or surgical bypass of any segment of the target vessel, including the target lesion (18). All patients were followed for 5 years and received optimal guideline-based medical therapy during follow-up.

### Statistical analysis

Continuous variables are expressed as the mean ± standard deviation (SD) or median [interquartile range (IQR)]. Categorical variables are expressed as numbers (percentages). Continuous variables were compared by ANOVA or the Kruskal‒Wallis test, and categorical variables were compared by chi‒squared analysis. The association between the ΔQFR and 5-year TVF in the five groups was estimated by the Kaplan‒Meier method and compared by the log-rank test. Restricted cubic spline regression analysis was used to assess the association between the ΔQFR and the hazard ratio (HR) for TVF. A 2-sided *P* value < 0.05 was considered to indicate statistical significance. All analyses were performed with R software version 4.1.1 (R Foundation for Statistical Computing, Vienna, Austria) and SPSS version 26 (IBM, Inc., New York, NY, USA).

## Results

### Study population

**Figure 1:**
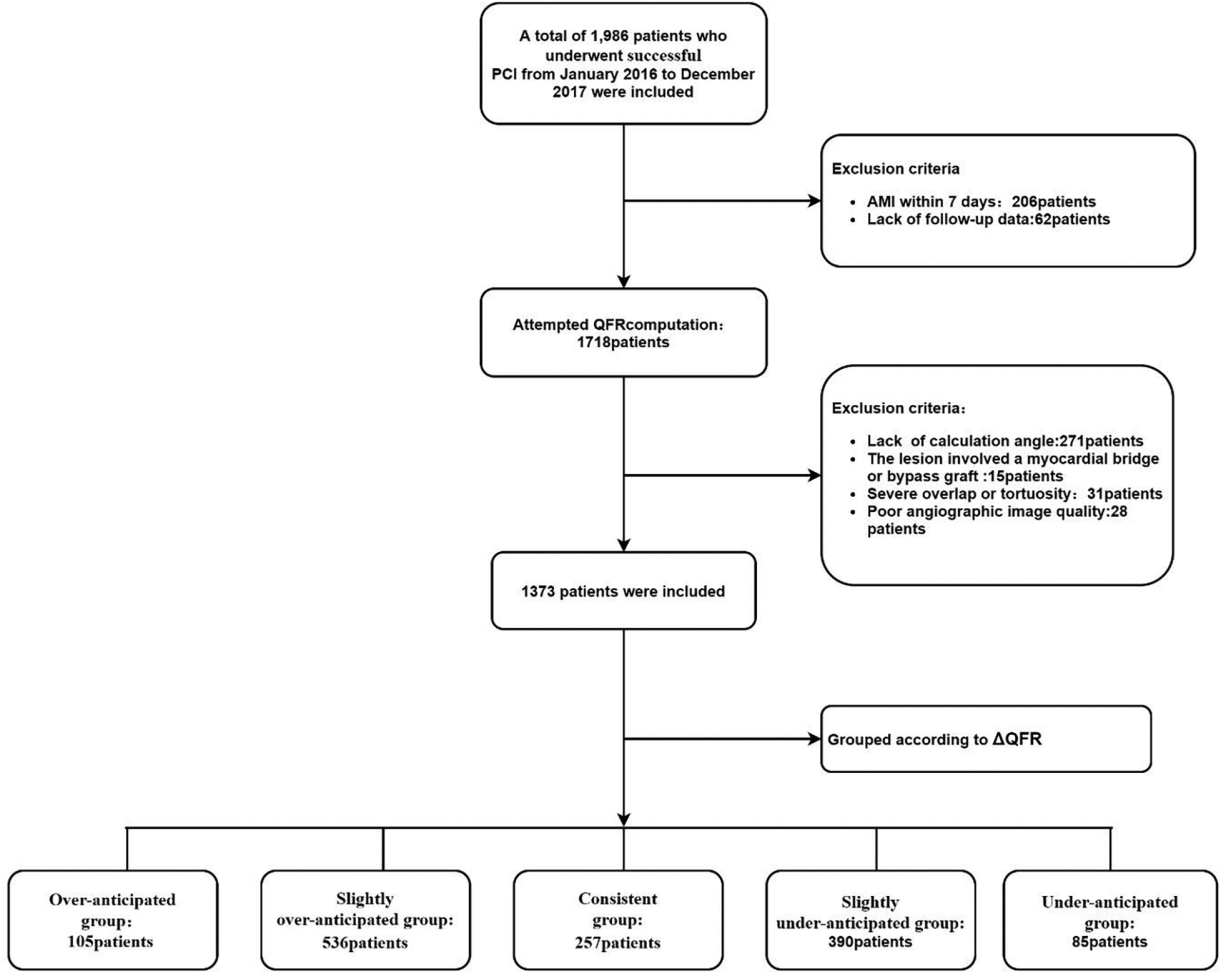
Study flowchart. PCI, percutaneous coronary intervention; STEMI, ST-segment elevation myocardial infarction; QFR, quantitative flow ratio; ΔQFR = post-PCI QFR—residual QFR.

Between January 1, 2016, and December 31, 2017, 1,986 patients who underwent PCI were screened for enrollment; 268 patients were excluded due to meeting the clinical exclusion criteria, and 345 patients were excluded due to meeting the angiographic exclusion criteria. The remaining 1,373 patients were included in the final analysis. According to the ΔQFR, all enrolled patients were further divided into five groups: (1) the overanticipated group: ΔQFR ≥ 0.1, *n* = 105; (2) the slightly overanticipated group: 0 < ΔQFR < 0.1, *n* = 536; (3) the consistent group: ΔQFR = 0, *n* = 257; (4) the slightly underanticipated group: −0.1 < ΔQFR < 0, *n* = 390; and (5) the underanticipated group: ΔQFR ≤ −0.1, *n* = 85. (Figure 1).

### Clinical baseline characteristics

The baseline characteristics are shown in Table 1. No significant differences were found between the groups with regard to age, sex, smoking status, hypertension, diabetes, previous MI, previous PCI, type of coronary artery disease, or post-PCI medication. Patients in the five groups had similar pre-PCI results for troponin I, LDL-C, NT-proBNP, blood glucose, serum creatinine, LVEF and E/E′.

**Table 1:** Baseline characteristics.

	Total (*N* = 1,373)	Overanticipated group (*N* = 105)	Slightly overanticipated group (*N* = 536)	Consistent group (*N* = 257)	Slightly underanticipated group (*N* = 390)	Underanticipated group (*N* = 85)	*P* value
Age (years)	65.05 ± 10.43	66.38 ± 11.26	65.04 ± 10.65	64.68 ± 10.16	64.67 ± 10.08	66.31 ± 10.16	0.438
Male, *n* (%)	1,057 (77.0)	82 (77.0)	408 (76.1)	193 (75.1)	309 (79.2)	65 (76.5)	0.747
Hypertension, *n*(%)	925 (67.4)	77 (73.3)	353 (65.9)	169 (65.8)	270 (69.2)	56 (65.9)	0.515
Diabetes, *n* (%)	472 (34.4)	41 (39.0)	179 (33.4)	80 (31.1)	137 (35.1)	35 (41.2)	0.369
Previous MI, *n* (%)	109 (7.9)	5 (4.8)	37 (6.9)	27 (10.5)	33 (8.5)	7 (8.2)	0.318
Previous PCI, *n* (%)	177 (12.9)	14 (13.3)	68 (12.7)	30 (11.7)	52 (13.3)	13 (15.3)	0.927
Smoking history, *n* (%)	722 (52.6)	52 (49.5)	283 (52.8)	130 (50.6)	213 (54.6)	44 (51.8)	0.829
Type of coronary artery disease							0.679
Unstable angina, *n* (%)	782 (57.0)	58 (55.2)	303 (56.5)	158 (61.5)	222 (56.9)	41 (48.2)	
Stable angina, *n* (%)	142 (10.3)	11 (10.5)	58 (10.8)	27 (10.5)	38 (9.7)	8 (9.4)	
NSTEMI, *n* (%)	251 (18.3)	23 (21.9)	100 (18.7)	37 (14.4)	69 (17.7)	22 (25.9)	
STEMI (≥7 days), *n* (%)	198 (14.4)	13 (12.4)	75 (14.0)	35 (13.6)	61 (15.6)	14 (16.5)	
Medications at discharge							
Antiplatelet agent, *n* (%)	1,371 (99.9)	105 (100.0)	536 (100.0)	256 (99.6)	389 (99.7)	85 (100.0)	0.656
Statin, *n* (%)	1,348 (98.2)	103 (98.2)	529 (98.7)	248 (96.5)	385 (98.7)	83 (97.6)	0.229
ACEI/ARB, *n*(%)	929 (67.7)	71 (67.6)	369 (68.8)	176 (68.5)	258 (66.2)	55 (64.7)	0.884
Troponin I(ug/L)	4.15 ± 10.90	2.44 ± 7.04	3.70 ± 10.01	4.39 ± 11.49	5.01 ± 12.18	4.10 ± 11.84	0.669
NT-proBNP (pg/ml)	191.5 (62.0,792.3)	347.5 (110.5,1,123.0)	185.0 (58.0,700.0)	160.0 (53.0,675.0)	198.0 (67.0,845.8)	220.0 (58.5,873.3)	0.758
Glucose (mmol/L)	6.34 ± 2.46	6.32 ± 2.10	6.46 ± 2.69	6.06 ± 2.18	6.33 ± 2.39	6.43 ± 2.46	0.313
Creatinine (umol/L)	86.65 ± 56.11	91.93 ± 31.65	87.95 ± 66.60	81.76 ± 45.55	86.84 ± 56.18	85.88 ± 29.23	0.530
LDL-C (mmol/L)	2.84 ± 1.03	2.89 ± 1.09	2.84 ± 1.04	2.86 ± 1.04	2.79 ± 1.02	2.89 ± 0.97	0.835
LVEF (%)	60.21 ± 11.37	60.09 ± 13.37	60.87 ± 10.66	60.75 ± 11.48	59.35 ± 11.46	58.29 ± 12.09	0.146
E/E′	13.63 ± 5.70	14.53 ± 6.32	13.45 ± 5.55	13.60 ± 6.01	13.67 ± 5.62	13.66 ± 5.33	0.563

The values are presented as the mean ± standard deviation, median (interquartile range) or *n* (%).

NSTEMI, Non-ST elevation myocardial infarction; STEMI, ST-segment elevation myocardial infarction; MI, myocardial infarction; PCI, percutaneous coronary intervention; ACEI, angiotensin-converting-enzyme inhibitor; ARB, angiotensin II receptor blocker; NT-proBNP, N-terminal pro-B type natriuretic peptide; LDL-C, low-density lipoprotein cholesterol; LVEF, left ventricular ejection fraction; E/E′, ratio of early diastolic mitral flow velocity to early diastolic mitral ring motion velocity.

### QCA and QFR analysis

The results of the QCA and QFR analyses are summarized in Tables 2, 3. In terms of target vessel locations, the consistent group had a greater proportion of LCX (22.6%) and a lower proportion of LAD (47.5%). Compared with those in the consistent group, the patients in the overanticipated group had longer stents (43.42 ± 17.47 vs. 31.41 ± 14.12, *P *< 0.001), more stents (1.65 ± 0.62 vs. 1.23 ± 0.45, *P *< 0.001), and similar stent diameters (3.03 ± 0.42 vs. 3.11 ± 0.44, *P *= 0.056).

**Table 2:** The results of the QCA and QFR analysis.

	Total (*N* = 1,373)	Overanticipated group (*N* = 105)	Slightly overanticipated group (*N* = 536)	Consistent group (*N* = 257)	Slightly underanticipated group (*N* = 390)	Underanticipated group (*N* = 85)	*P* value
Target vessel							0.015
LAD, *n* (%)	794	69 (65.7)^b^	319 (59.5)^b^	122 (47.5)^a^	232 (59.5)^b^	52 (61.2)^a^	
LCX, *n* (%)	223	15 (14.3)^a,b^	75 (14.0)^b^	58 (22.6)^a^	64 (16.4)^a,b^	11 (12.9)^a,b^	
RCA, *n* (%)	356	21 (20.0)^a^	142 (26.5)^a^	77 (30.0)^a^	94 (24.1)^a^	22 (25.9)^a^	
Pre-PCI							
DS (%)	57.51 ± 12.09	56.61 ± 11.52	56.88 ± 12.52	57.94 ± 12.50	58.13 ± 11.89	58.36 ± 9.36	0.433
AS (%)	77.44 ± 11.83	76.58 ± 10.96	76.91 ± 11.75	76.61 ± 11.83	78.51 ± 12.49	79.51 ± 9.83	0.069
MLD (mm)	1.15 ± 0.59	1.10 ± 0.39	1.13 ± 0.43	1.15 ± 0.47	1.19 ± 0.85	1.17 ± 0.47	0.535
QFR	0.68 ± 0.18	0.59 ± 0.13	0.70 ± 0.16	0.73 ± 0.16	0.65 ± 0.19	0.56 ± 0.20	<0.001
Residual QFR	0.96 ± 0.05	0.84 ± 0.04	0.95 ± 0.04	0.99 ± 0.03	0.97 ± 0.03	0.96 ± 0.06	<0.001
Post-PCI							
DS (%)	25.70 ± 10.74	23.63 ± 8.75	21.83 ± 8.35	20.44 ± 8.21	31.26 ± 8.84	43.10 ± 11.88	<0.001
AS (%)	38.38 ± 16.37	33.70 ± 13.53	32.44 ± 14.13	29.68 ± 13.74	47.90 ± 11.04	64.28 ± 12.44	<0.001
MLD (mm)	2.02 ± 0.54	1.97 ± 0.40	2.09 ± 0.50	2.20 ± 0.51	1.90 ± 0.55	1.58 ± 0.57	<0.001
QFR	0.96 ± 0.06	0.98 ± 0.03	0.98 ± 0.03	0.99 ± 0.03	0.94 ± 0.04	0.79 ± 0.10	<0.001

The values are presented as the mean ± standard deviation (*n*%).

PCI, percutaneous coronary intervention; LAD, left anterior descending coronary artery; LCX, left circumflex coronary artery; RCA, right coronary artery; AS, area stenosis; DS, diameter stenosis; MLD, minimal lumen diameter; QFR, quantitative flow ratio.

Each letter represents a subset of the group based on the difference level between residual QFR and post-PCI QFR, the same letter means no significant, with no significant difference between groups at *p* = 0.05 level.

**Table 3:** Parameters related to the stent.

	Overanticipated group (*N* = 105)	Slightly overanticipated group (*N* = 536)	Consistent group (*N* = 257)	Slightly underanticipated group (*N* = 390)	Underanticipated group (*N* = 85)	*P* value
Stent diameter(mm)	3.03 ± 0.42	3.03 ± 0.41	3.11 ± 0.44	3.03 ± 0.41	2.96 ± 0.41	0.023
Stent length(mm)	43.42 ± 17.47	32.86 ± 17.25	31.41 ± 14.12	30.80 ± 13.66	29.09 ± 15.70	<0.001
Number of stents	1.65 ± 0.62	1.32 ± 0.55	1.23 ± 0.45	1.25 ± 0.47	1.31 ± 0.49	<0.001

The values are presented as the mean ± standard deviation [*n* (%)].

There was a significant difference in the pre-PCI QFR among the five groups (*P *< 0.001), with lower pre-PCI QFRs in the overanticipated group and underanticipated group. Significant differences in the post-PCI QFR were found in the five groups, of which the underanticipated group had a significantly worse post-PCI QFR than the other groups. No significant differences in DS%, AS% or MLD were found among the five groups at pre-PCI, whereas a difference was found for the post-PCI between the groups.

### Clinical outcomes

A five-year follow-up was completed for all eligible patients, with a median follow-up of 61 months. Comparisons of the clinical outcomes between the 5 groups are shown in Table 4. TVF occurred within 5 years in 189 patients in all the groups; 14 patients were in the overanticipated group, 66 patients were in the slightly overanticipated group, 30 patients were in the consistent group, 60 patients were in the slightly underanticipated group, and 19 patients were in the underanticipated group. The consistent group had the lowest incidence of TVF, and the underanticipated group had the highest risk of TVF. Supplementary Table S1 compares the difference in the incidence of TVF between the consistent group and the other groups. Supplementary Table S2 showes shows that TVF is independent of target vessel distribution. The incidence of TVF was significantly greater in the underanticipated group than in the consistent group (*P *= 0.008), whereas no significant differences were found when comparing the underanticipated group with the other three groups.

**Table 4:** Clinical outcomes at the 5-year follow-up.

	Overanticipated group (*N* = 105)	Slightly overanticipated group (*N* = 536)	Consistent group (*N* = 257)	Slightly underanticipated group (*N* = 390)	Underanticipated group (*N* = 85)	*P* value
TVF, *n* (%)	14 (13.3)^a,b^	66 (12.3)^b^	30 (11.3)^b^	60 (15.4)^a,b^	19 (22.4)^a^	0.076
Cardiovascular death, *n* (%)	6 (5.7)	30 (5.6)	14 (5.4)	25 (6.4)	3 (3.5)	0.885
MI, *n* (%)	1 (1.0)	11 (2.1)	2 (0.8)	5 (1.3)	3 (3.5)	0.363
TVR, *n* (%)	7 (6.7)^a,b^	32 (6.0)^a^	15 (5.8)^a^	33 (8.5)^a,b^	13(15.3)^b^	0.025

The values are presented as *n* (%).

PCI, percutaneous coronary intervention; TVF, target vessel failure. MI, myocardial infarction; TVR, target vessel revascularization.

Each letter represents a subset of the group based on the difference level between residual QFR and post-PCI QFR, the same letter means no significant, with no significant difference between groups at *p* = 0.05 level.

**Figure 2:**
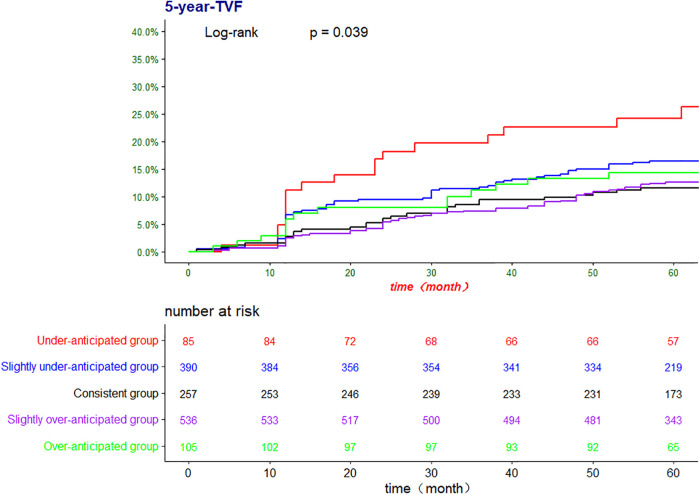
The Kaplan-Meier analysis for TVF according to ΔQFR. TVF, target vessel failure; QFR, quantitative flow ratio; ΔQFR = post-PCI QFR—residual QFR.

**Figure 3:**
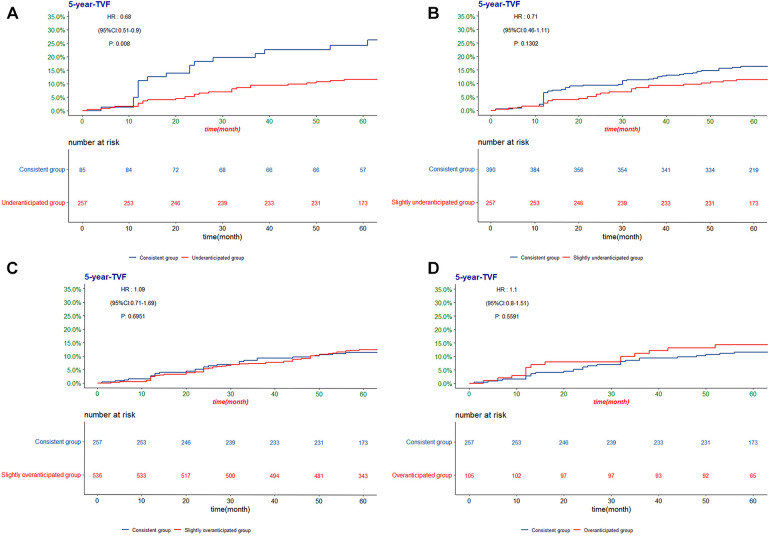
The Kaplan-Meier analysis of TVF according to the difference between post-PCI QFR and residual QFR for the (**A**) consistent group and underanticipated group; (**B**) consistent group and slightly underanticipated group; (**C**) consistent group and slightly overanticipated group; (**D**) consistent group and overanticipated group. TVF, target vessel failure; QFR, quantitative flow ratio.

The Kaplan‒Meier method was used to further confirm the difference in the incidence of TVF among the five groups (Figures 2, 3). There was a significant difference in the risk of 5-year TVF among the five groups (log-rank *P *= 0.039). The incidence of TVF was lower in the consistent group than in the underanticipated group (HR = 0.068, 95% CI = 0.51–0.90, *P *= 0.008), while no significant differences were found between the consistent group and the remaining three groups.

**Figure 4:**
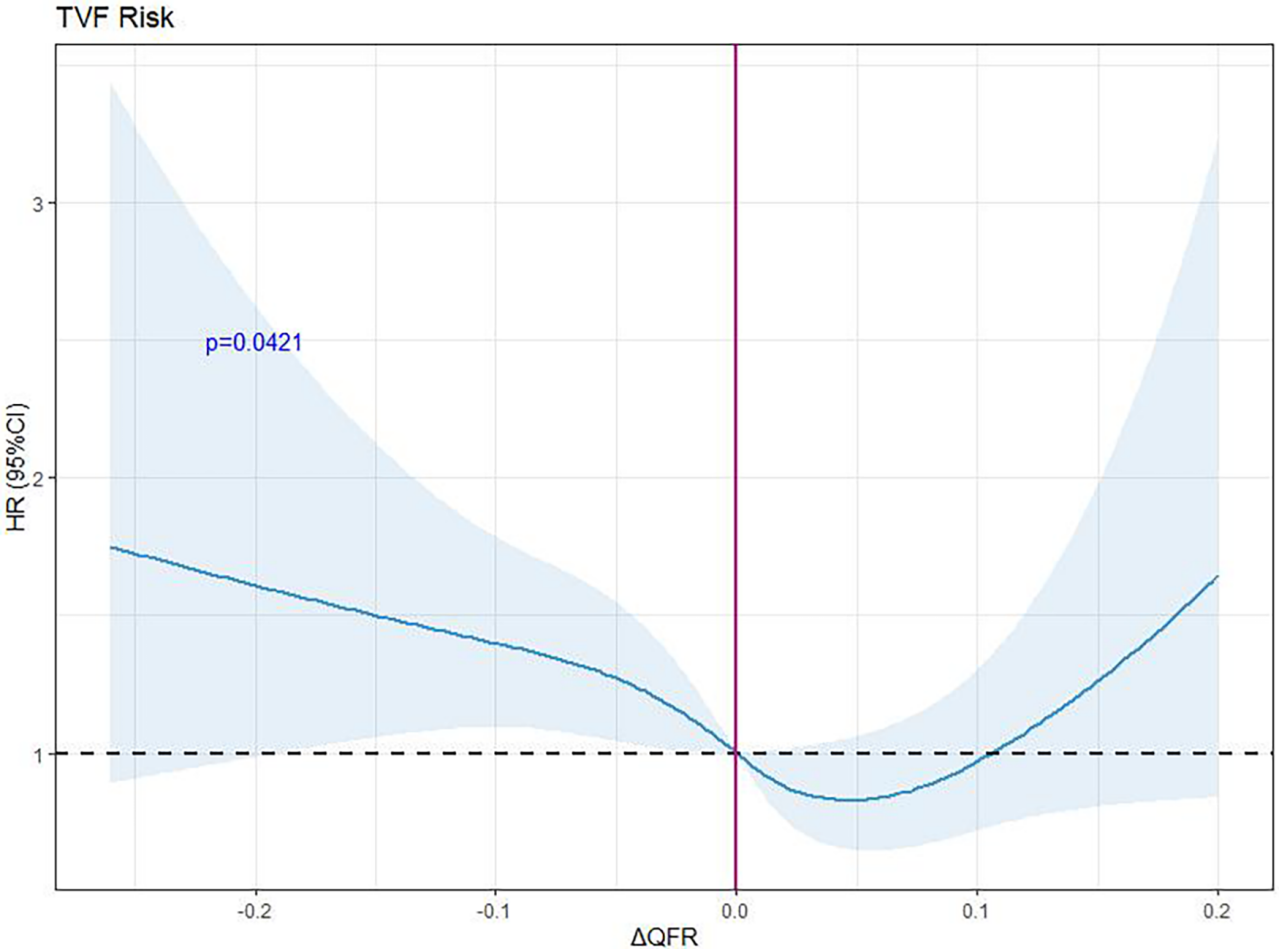
Hazard ratios for the TVF based on restricted cubic spline. The purple line represents the reference hazard ratio, and the blue area represents the 95% confidence interval. RCS, restricted cubic spline; HR, hazard ratio; ΔQFR, postoperative QFR minus preoperative residual QFR.

Restricted cubic spline regression analysis was used to analyze and visualize the association between ΔQFR and TVF (Figure 4). As the ΔQFR increased, the hazard ratio of TVF first decreased and then gradually increased.

### Predictive performance of the ΔQFR for 5-year TVF

Univariate analysis and multivariate Cox regression analysis were performed to evaluate the predictive performance of the ΔQFR for the 5-year TVF (Table 5). After screening via univariate Cox regression (*P *< 0.05), ΔQFR ≤ −0.1, age, previous MI, hypertension, diabetes, creatinine, and LVEF were included in the multivariate analysis. The multivariate analysis revealed that a ΔQFR ≤ −0.1, old age, previous MI, and diabetes were independent risk factors for TVF, and a high LVEF was an independent protective factor.

**Table 5:** Univariate and multivariate Cox regression analyses of factors influencing the TVF.

	Univariable		Multivariable	
	OR (95% CI)	*P* value	OR (95% CI)	*P* value
Age	1.023 (1.009–1.038)	0.001	1.019 (1.005–1.034)	0.014
Male	0.987 (0.704–1.383)	0.940		
Smoking history	1.027 (0.771–1.368)	0.856		
Previous MI	2.518 (1.712–3.701)	<0.001	1.689 (1.119–2.550)	0.013
Hypertension	1.398 (1.009–1.936)	0.044	1.246 (0.884–1.754)	0.209
Diabetes	2.216 (1.664–2.951)	<0.001	1.842 (1.366–2.484)	<0.001
ΔQFR ≤ −0.1	1.874 (1.166–3.012)	0.009	1.673 (1.039–2.698)	0.034
Troponin I	0.995 (0.973–1.017)	0.632		
LDL-C	0.916 (0.793–1.059)	0.233		
LVEF	0.968 (0.958–0.979)	<0.001	0.975 (0.964–0.986)	<0.001

ΔQFR = post-PCI QFR minus residual QFR.

PCI, percutaneous coronary intervention; TVF, target vessel failure; MI, myocardial infarction; LDL-C, low-density lipoprotein cholesterol; LVEF, left ventricular ejection fraction; QFR, quantitative flow ratio.

## Discussion

The present study was the first to evaluate the prognostic value of the consistency between the residual QFR and post-PCI QFR in TVF. The main findings are as follows: (1) The incidence of TVF in the consistent group was significantly lower than that in the underanticipated group, whereas it was similar to that in the overanticipated group, suggesting that the consistency between the residual QFR and post-PCI QFR is associated with the long-term prognosis of patients. (2) This study provides a new perspective on the residual QFR to further explore the potential of the QFR in clinical practice.

Despite successful revascularization, some patients with CAD still experience symptoms of angina pectoris or recurrent cardiovascular adverse events (19, 20). Previous studies suggest that plaque burden rather than stenosis is one of the main predictors of cardiovascular adverse events (21), which may partly explain the uncertain association between the degree of luminal stenosis and the severity of myocardial ischemia. Previous landmark studies have demonstrated the instrumental value of the QFR in guiding the PCI procedure and further improving the clinical prognosis (10–12). The residual QFR is a predicted QFR value based on coronary angiographic imaging that simulates successful stent implantation in the culprit lesion, correlates well with the post-PCI FFR and QFR, and predicts the occurrence of adverse events after revascularization (13, 14, 16, 22). A retrospective analysis of the PANDA III trial showed that the predicted clinical outcome of residual QFR-guided PCI was superior to that of angio-guided PCI (15, 23). In addition, the ability of the residual QFR to distinguish functional stenosis was confirmed (16). Compared with the post-PCI QFR, the residual QFR can predict post-PCI coronary function in advance and provide anticipated post-PCI vascular information on the culprit lesion segment, further delaying revascularization in lesions with anticipated insignificant functional benefit. The number and length of stents that are assigned to be implanted in the coronary arteries can be reduced by knowing the stenoses with relatively high treatment benefits and the coronary lesions with potentially low treatment benefits in the index PCI.

In our study, the post-PCI QFR was significantly lower than the residual QFR in the underanticipated group, with a greater incidence of TVF than in the consistent group (22.4% vs. 11.3%, *P *= 0.003). A multivariate Cox regression model revealed that a ΔQFR ≤ −0.1 (OR: 1.673, 95% CI: 1.039–2.698 *P *= 0.034) was an independent risk factor for TVF, which indicates the potential for consistency between the residual QFR and post-PCI QFR to predict adverse events; namely, a QFR significantly lower than the residual QFR is prone to be associated with TVF after successful revascularization. Accumulating evidence suggests that a poor physiological outcome may be indicative of stent malapposition, an uncovered stent, a stent under expansion, or incomplete postdialation (24). The residual QFR was calculated as the maximum QFR outside the stent segment of the entire vessel, which may lead to an inadequate assessment of stent malapposition or under expansion. A suboptimal stenting strategy may increase in-stent restenosis and endothelial hyperplasia (25), further increasing the incidence of repeat revascularization, which may explain the high incidence of TVF in the underanticipated group. The stent diameter in the underanticipated group in this study was smaller (2.96 ± 0.41 vs. 3.11 ± 0.44, *P *= 0.004) than that in the consistent group, supporting the previous hypothesis. A low residual QFR suggests that the target vessel may have a limited or diffuse lesion that the operator is unaware of or that the benefit of intervention for this coronary lesion is low, and this information may help to modify the PCI strategy (14). In addition, compared with the consistent group, the overanticipated group had a greater mean number of stents implanted (1.65 ± 0.62 vs. 1.23 ± 0.45, *P *< 0.001) and a longer total stent length (43.42 ± 17.47 vs. 30.80 ± 13.66, *P *< 0.001), which may have contributed to the greater post-PCI QFR than residual QFR in the overanticipated group. According to the RCS regression analysis, the risk of VTVF was nonlinearly related to the ΔQFR and had a V-shape. Although patients in the underanticipated group had a higher risk of TVF, the incidence of TVF was not reduced in the overanticipated patients. This may indicate that a more aggressive PCI strategy leads to a higher post-PCI QFR but prolongs the operation time, and too many stents may increase stent-related risks. Furthermore, the post-PCI QFR was significantly greater than the residual QFR in the overanticipated group, while the incidence of TVF was not lower than that in the consistent group (13.3% vs. 11.3%, *P *= 0.559), which confirms the ability of the residual QFR to discriminate less functionally beneficial coronary lesions and indicates that aggressive treatment does not reduce the incidence of adverse events in such lesions.

This study has several limitations. First, the current study was a single-center, retrospective, observational study. These findings need to be further validated by prospective, multicenter studies. Second, some patients were excluded due to the lack of optimal angiographic images for QFR analysis, which led to selection bias. In addition, the residual QFR is a novel index that provides vascular information for PCI treatment, but the accuracy and feasibility of a treatment strategy based on the residual QFR need to be further confirmed.

## Conclusions

The consistency between the residual QFR and post-PCI QFR may be associated with the long-term prognosis of patients. Patients whose post-PCI QFR is significantly lower than the residual QFR may be at greater risk of TVF. An aggressive PCI strategy for lesions anticipated to have less functional benefit may not result in a better clinical outcome.

## Data Availability

The raw data supporting the conclusions of this article will be made available by the authors, without undue reservation.
