# Supplementary material for: Predictive value of the inconsistency between the residual and post-PCI QFR for prognosis in PCI patients
Source: Front Cardiovasc Med. 2024 Apr 17;11:1297218. doi: 10.3389/fcvm.2024.1297218 (PMC11062415; doi:10.3389/fcvm.2024.1297218)
Supplement: Supplementary file 1 [file Datasheet1.docx]

**Supplementary Table 1. Comparison of TVF and TVR between the Consistent group and other group**

|  | Consistent group vs. Over-anticipated group | Consistent group vs. Slightly over-anticipated group | Consistent group vs. Slightly under-anticipated group | Consistent group vs. Under-anticipated group |
| --- | --- | --- | --- | --- |
| TVF | 0.559 | 0.695 | 0.130 | 0.008 |
| TVR | 0.764 | 0.941 | 0.213 | 0.006 |

PCI, percutaneous coronary intervention; TVF, target vessel failure. MI, myocardial infarction; TVR, target vessel revascularization.

**Supplement table2.target vessel failure* target vessels**

|  |  | TVF | NO TVF | χ^2^ | P |
| --- | --- | --- | --- | --- | --- |
| target vessels | LAD | 685 | 109 | 4.384 | 0.112 |
|  | LCX | 201 | 22 |  |  |
|  | RCA | 299 | 57 |  |  |

TVF, target vessel failure. LAD, left anterior descending coronary artery; LCX, left circumflex coronary artery; RCA, right coronary artery;
